# The analyses of human MCPH1 DNA repair machinery and genetic variations

**DOI:** 10.1515/med-2024-0917

**Published:** 2024-02-29

**Authors:** Oluwafemi G. Oluwole

**Affiliations:** Biomedical Research Centre, Nuffield Department of Medicine, Wellcome Centre for Human Genetics, University of Oxford, Oxford, OX3 7BN, UK; Division of Human Genetics, University of Cape Town, Cape Town, South Africa; Non-communicable Diseases Department, Institute of Primate Research, Nairobi, Kenya

**Keywords:** DNA repair, variants, MCPH1, complex interactions

## Abstract

Causal mutations in the MCPH1 gene have been associated with disorders like microcephaly, and recently congenital hearing impairment. This study examined the MCPH1 DNA repair machinery and identified genetic variations of interest in gnomAD database to discuss the biological roles and effects of rare variants in MCPH1-related diseases. Notably, MCPH1 coordinates two of the seven known mechanisms of DNA repair which confirmed its roles in neurogenesis and chromatin condensation. A pathogenic missense variant in MCPH1 p.Gly753Arg, and two pathogenic frameshifts MCPH1 p.Asn189LysfsTer15 and p.Cys624Ter identified in this study, already had entries in ClinVar and were associated with microcephaly. A pathogenic frameshift in MCPH1 p.Val10SerfsTer5 with a loss-of-function flag and a pathogenic stop gained p.Ser571Ter variants with ultra-rare allele frequency (MAF ≤ 0.001) were identified but have not been linked to any phenotype. The predicted pathogenic ultra-rare variants identified in this study, warranty phenotypic discovery, and also positioned these variants or nearby deleterious variants candidate for screening in MCPH1-associated rare diseases.

## 1 Introduction

**Figure 1:**
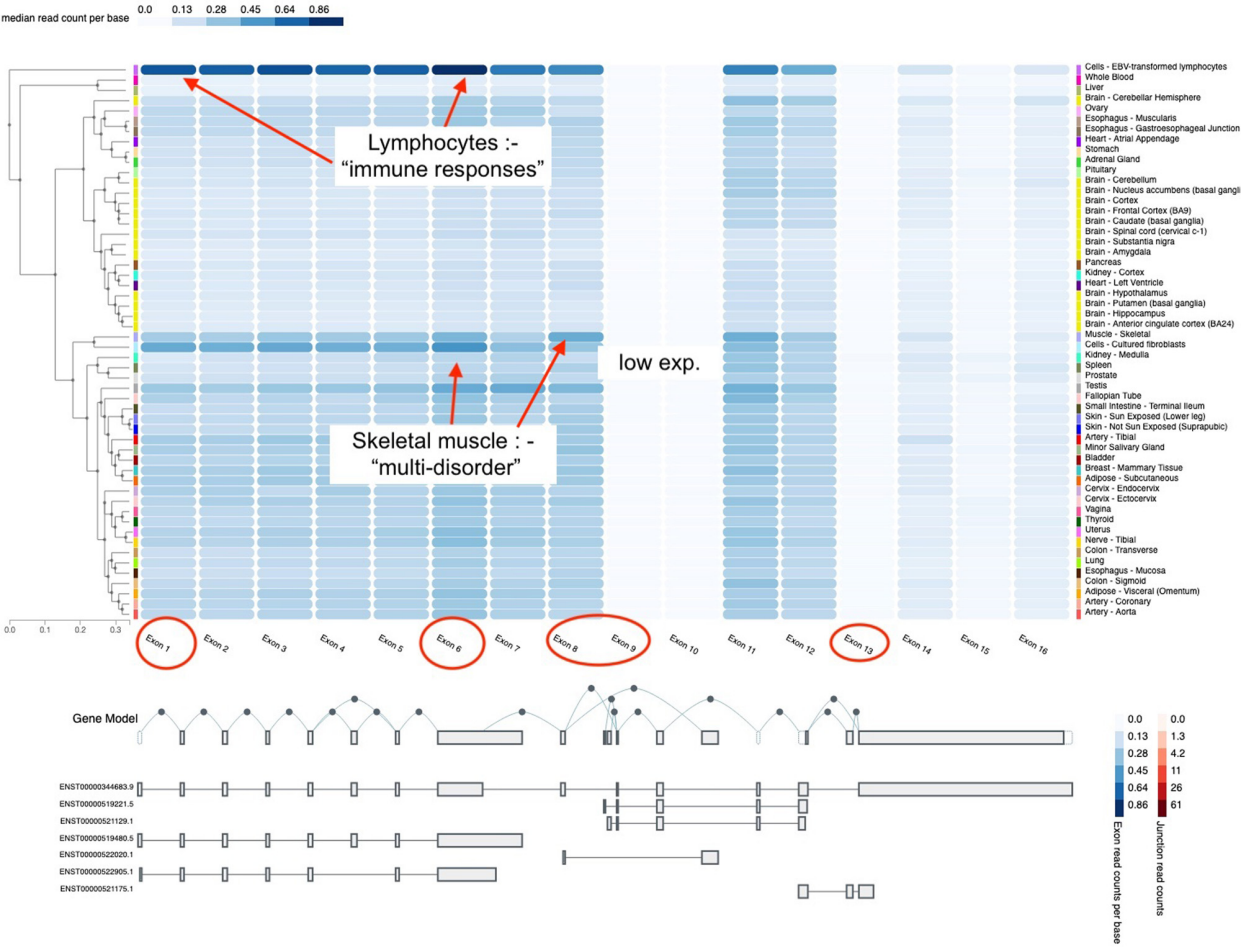
Annotation of Exon Expression of MCPH1 derived from the GTEx Analysis V8. The functional annotations of the rare variants p.Val10SerfsTer5 andp.Ser571Ter to the exons and tissue specific expression values.

**Figure 2:**
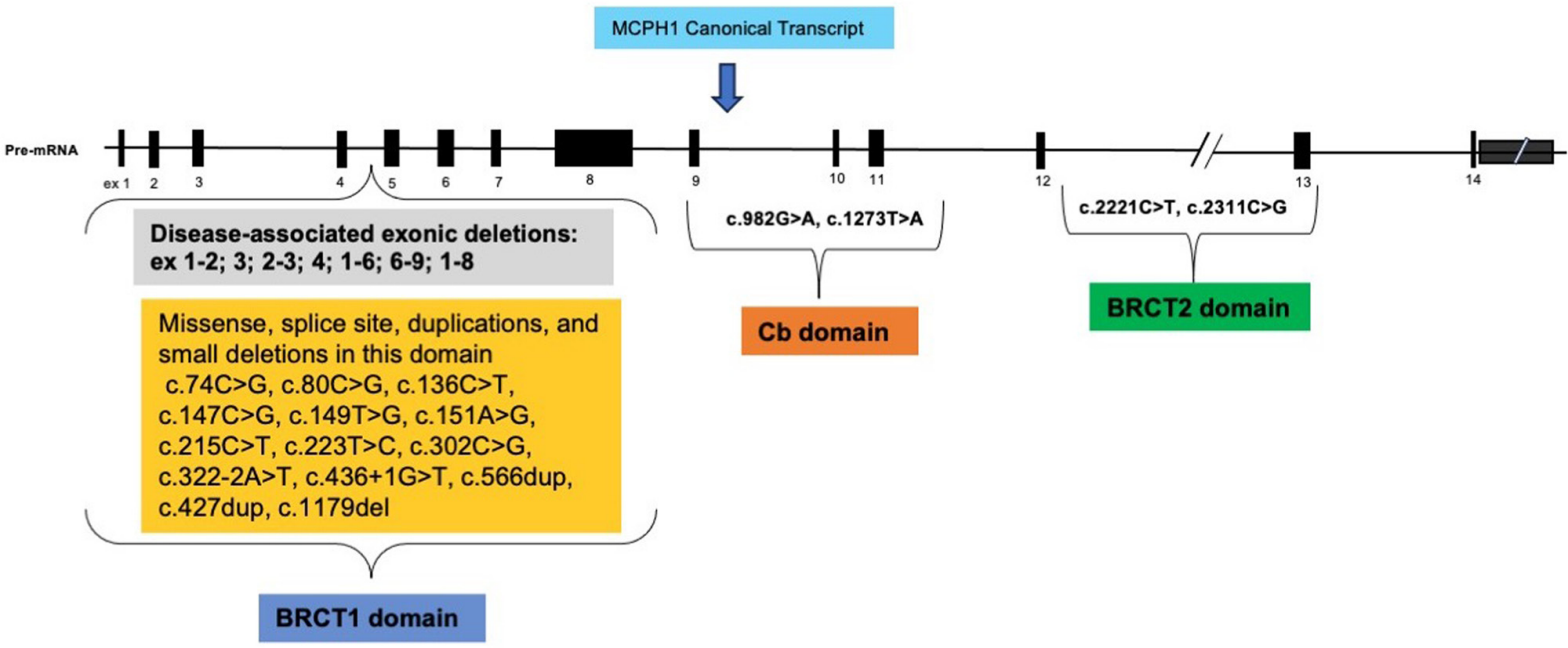
Overview of MCPH1 disease-associated variants identified in different domains of the canonical transcript. (ENST00000344683.9)

The human MCPH1 gene is ubiquitously expressed in adult tissues including the blood, brain, testes, pancreas, and liver (Figure 1). The MCPH1 gene is highly conserved in many species [1]. The MCPH1 is a DNA damage response protein involved in the regulation of CHK1 and BRCA1 [2]. It has been implicated in chromosome condensation and DNA damage-induced cellular responses [3,4]. It plays a role in neurogenesis by coordinating the cell cycle and the centrosome cycle [3,5]. The human MCPH1 contains 14 coding exons. The BRCT1, BRCT2, and BRCT3 domains of MCPH1 have high mutational hotspots (Figure 2). The BRCT2/3 domains of the human MCPH1 gene are crucial and interact with phosphorylated residues and with different subunits of condensin II to regulate chromosome condensation [4]. The *MCPH1* regulation of the DNA repair mechanisms maintains the genome integrity.

MCPH1 has been strongly associated with primary microcephaly and other neurological diseases like epilepsy, learning disabilities, speech delays, and infertility [6–8]. More recently, mutations in the MCPH1 were associated with congenital hearing impairment (CHI) [1,9], and previously in otitis media in the mice model [10]. The study on otitis media showed a knockout mouse exhibiting mild to moderate hearing impairment with a penetrance level of 70% in which the mice suffered from otitis media [10]. The study on CHI was the first to identify an associated homozygous missense mutation in MCPH1 likely to explain the cause of CHI in a Cameroonian family [1,9]. The role of MCPH1 in the regulation of mitochondrial activity and metabolism was earlier reported [4], suggesting that MCPH1 is associated with various biological processes and could have a pleiotropic role. For example, microcephaly deafness syndrome is an extremely rare genetic disorder that consists of microcephaly, CHI, mild intellectual disability, speech delay, low height, and facial dysmorphisms. The facial dysmorphisms and hearing loss (HL) levels in microcephaly are very similar to what we previously reported [11–13]

MCPH1 protein is co-localized in the centrosome and associated with mitochondria in mice and humans [6,14]. The protein levels of MCPH1 are tightly regulated and prolonged overexpression of MCPH1 seems to be toxic to the cell [3,15,16]. Various studies on MCPH1’s mechanistic and genetic functions can shed more light on the roles and biological functions of this gene. The present study aimed to analyse the frequencies of genetic variants in MCPH1 in the largest publicly available genotypic variations data and to further understand the possible impact of disease causing variations to its biological activities.

## 2 Methods

### 2.1 Re-analysing the genetic variations in *MCPH1*

The analyses of a large set of variations identified in MCPH1 were performed by extracting the MCPH1 data in The Genome Aggregation Database gnomAD [17] v2.1.1 data set (GRCh37/hg19) which provided 125,748 exome sequences and 15,708 whole-genome sequences from unrelated individuals sequenced as part of various disease-specific and population genetic studies. The gene structure and information were derived in Ensembl (https://www.ensembl.org) and the chromosome location and regional details for MCPH1 were mined. Chromosome 8 where the MCPH1 is located was downloaded on gnomAD into a local machine for further analyses.

### 2.2 Data extractions

*The file was downloaded with a command:*

wget https://storage.googleapis.com/gcp-public-data--gnomad/release/2.1.1/vcf/exomes/gnomad.exomes.r2.1.1.sites.8.vcf.bgz

*The file was converted from bgz to gz with a command:*

mv gnomad.exomes.r2.1.1.sites.8.vcf.bgz gnomad.vcf.gz

*The gz file was decompressed with the command:*

gzip -d gnomad.vcf.gz

*With the grep command, the MCPH1 data were extracted from the decompressed vcf with a command.*

grep MCPH1 gnomad.vcf > onlyMCPH1.vcf

*The number of homozygote and heterozygote variants was determined with the command:*

cat onlyMCPH1.vcf|awk -v OFS =”\t” ‘$0!∼ “^#“ {hom_ref = 0; hom_alt = 0; het = 0; for(*i* = 10;*i* < = NF; *i*++) { if($i ∼/0\|0/) hom_ref++; else if($i ∼/1\|1/) hom_alt++; else het++;} print $1, $2, hom_ref, hom_alt, het}’ > allelecount

The biallelic variants that PASS (position has passed all filters, i.e., a call is made at this position) and adequate read depth at this position, then, the filters-exome and filters-genome were extracted and the output file was used as input for variant effect predictor (VEP) for pathogenicity predictions. The clinical significance (ClinVar) information about each variant was retrieved as well. The CSV file was analyzed statistically using the R_Shiny_apps and Python 3.

### 2.3 Understanding the MCPH1 mechanistic functions

For a detailed understanding of the pathways and biological relevance of MCPH1, the human MCPH1 was queried in Reactome [18–21] (https://reactome.org) and GTEx [22] (https://gtexportal.org) databases. The DNA repair was selected in the search menu. Then, MCPH1 was queried for each of the seven known terms under DNA repair to identify which of the pathway(s) has entries for MCPH1. Furthermore, the tissue expression profile of MCPH1 was determined in the GTEx database accordingly. The biological domains with molecular, cellular, and biological processes representing the current information on the biological functions of *MCPH1* were investigated in the Gene Ontology [23,24] database.

## 3 Results

MCPH1 has been reported extensively localized in centrosomes. MCPH1 can co-localize in the nucleoplasm; however, the function of MCPH1 localization in nucleoplasm is not known. Further analyses of the terms in gene ontology functions were matched to the processes and functions in the nuclear and intracellular lumen, which may suggest the likelihood functions of MCPH1 localization in the nucleoplasm and in the major organelles that are found in the cellular component (Figure 1). The gene expression of MCPH1 is high in many tissues; the highest is in the blood (lymphocytes) and brain (Figure 1).

The variant analyses identified a total of 2706 variants in the MCPH1 variations data in gnomAD. Of these, 1709 variants PASS filter. The variants predicted likely pathogenic or pathogenic were selected and their distributions and matching to VEP annotation and ClinVar classifications were described in Table 1. Missense variants account for the majority of VEP annotation (Table 1). The identified pathogenic missense p.Gly753Arg, and two pathogenic frameshift p.Asn189LysfsTer15 and p.Cys624Ter had entries in ClinVar; however, the pathogenic frameshift p.Val10SerfsTer5 with loss-of-function flag and a pathogenic stop gained p.Ser571Ter have not been associated with a phenotype or documented in ClinVar.

**Table 1:** Protein altering variants in identified in MCPH1 in the gnomAD data, and their VEP and ClinVar annotations considered pathogenic, likely pathogenic or uncertain significance

Count of ClinVar	VEP								
ClinVar Significance	missense_variant	frameshift_variant	splice_region_variant	stop_gained	synonymous_variant	splice_acceptor_variant	5_prime_UTR_variant	splice_donor_variant	Grand total
Uncertain significance	56	1	3		3	1	1		65
Pathogenic	1	3		2					6
Likely pathogenic				1		1		1	3
Grand total	57	4	3	3	3	2	1	1	74

The graphical structure of the MCPH1 revealed the mutational hotspots (Figure 2). Figure 2 mapped some of the variants that have been identified and linked to diseases in the MCPH1 canonical transcript. Of note, the first variant (p.Val10SerfsTer5) identified in this study is in the BRCT1 domain. The second variant (p.Ser571fsTer5) is in the BRCT2 domain.

**Figure 3:**
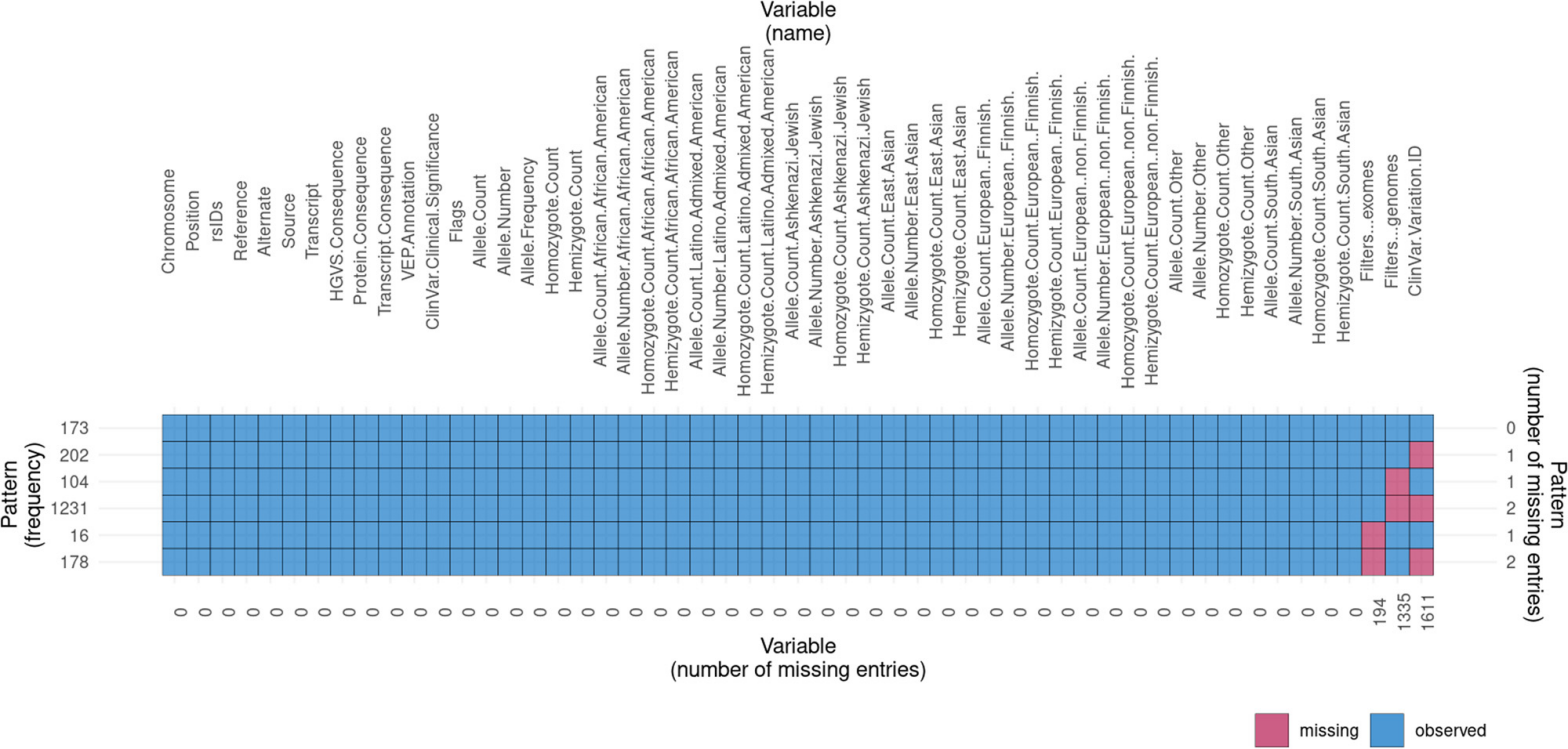
The missing data plot showing the number of missing entries and the pattern frequency observed in the gnomAD data analyzed in this study.

**Figure 4:**
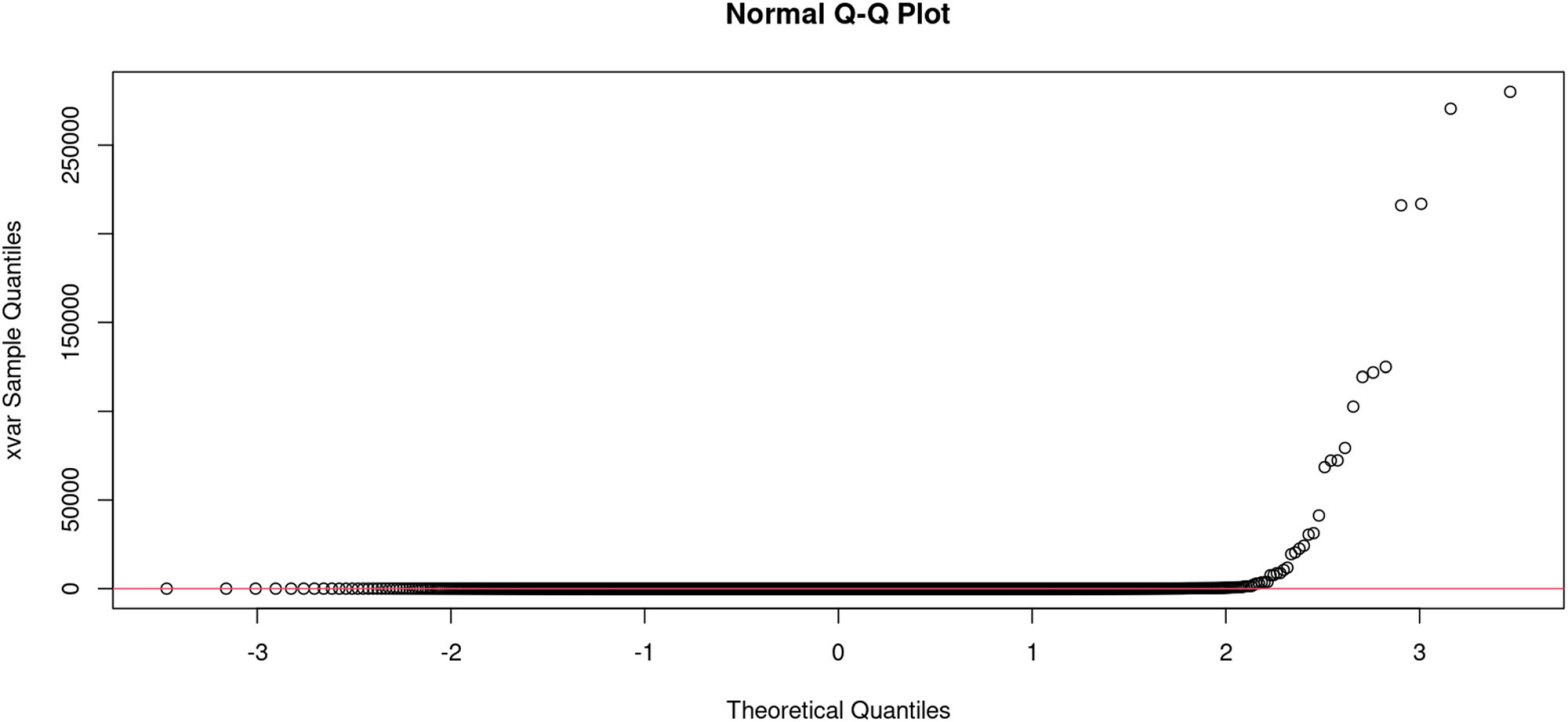
The normal *Q*–*Q* plot to check the assumption that the allele number per position is normally distributed, the data have more extreme values and are not normally distributed.

For protein-truncating variation, the assumption is that for tolerance loss of gene function: null (where the loss of both copies of the gene is tolerated), recessive (where the loss of a single copy of the gene is tolerated, but not loss of both copies), and haploinsufficient (where the loss of a single copy of the gene is not tolerated). As MCPH1 is an autosomal recessive gene, the variants might be disease-causing or tolerated. The estimates of missing data and the pattern of missing data observed in the MCPH1 gnomAD data were plotted (Figure 3). The *Q*–*Q* normality plot and Kolmogorov–Smirnov normality of allele count vs allele number indicate that the data are not normally distributed (Figure 4). While the Pearson’s Chi-squared test of allele count vs allele number per position had *X*-squared = 2256.2, degree of freedom (df) = 2,232, *p*-value = 0.3554, with the multiple *R*-squared values of 0.001778, adjusted *R*-squared value of 0.001253, *F*-statistic value of 3.388 on 1, and 1,902 DF, for *p*-value of 0.06583.

## 4 Discussion

Identifying very important genes and their phenotypic functions is a major goal in genetics. Genetic variations are major determinants of susceptibility to disease, response to therapy, and clinical outcomes. Advances in short-read sequencing technologies, despite some shortcomings, have enabled the identification of several genetic variants. The major challenge is how to adequately identify the pathogenic variants in sequenced data and their clinical interpretations. The gnomAD v2.1.1 data set (GRCh37/hg19) analyzed in this study provided 125,748 exome sequences and 15,708 whole-genome sequences from unrelated individuals sequenced as part of the largest various disease-specific and population genetic studies. Missing data can bias results [25]. In statistics, missing data, or missing values, occur when no data value is stored for the variable in an observation. Different sequencing platforms and variability in read depth or variant calling methods can introduce missingness [26]. Nonetheless, through the data cleaning, filtering steps, and the annotation for pathogenic variants in the MCPH1 gene in this study, a pathogenic missense p.Gly753Arg and two pathogenic frameshifts p.Asn189LysfsTer15 and p.Cys624Ter identified in this study already had entries in ClinVar and associated with microcephaly. A pathogenic frameshift p.Val10SerfsTer5 with a loss-of-function flag and a pathogenic stop gained p.Ser571Ter variants with very low allele frequency (MAF ≤ 0.001) were identified in the study but have not been associated with any phenotype, which suggests their screening potential in MCPH1 association syndrome.

The genetic locations for these variations are in the active domain and consensus transcripts of the MCPH1 gene. The BRCT1 is the most mutated and disease-associated domain in the MCPH1 gene (Figure 2). The first variant (p.Val10SerfsTer5) identified in this study is in the BRCT1 domain. The second variant (p.Ser571fsTer5) is in the BRCT2 domain. Only a few disease-associated variants have been identified in the BRCT2 active domain. New studies are emerging with reports about mutations identified in this domain. New studies are emerging with reports about mutations identified in this domain [1,9,27]. While there is no evidence to suggest what are the phenotypes the carriers of these variants exhibit, the VEP pathogenicity predictions in this study strongly suggest deleterious. The MCPH1 gnomAD constraint metrics (o/e 1.63 and 1.11, respectively) for missense and loss-of-function variants suggest intolerance. Several inherited diseases have been linked to gene-truncating variants in the Online Mendelian Inheritance in Man (OMIM) database. The graphical view of the MCPH1 gene showed that these variants were at positions leading to the loss of a functional domain. Currently, missense variants are the most reported forms of variations in the MCPH1 gene. However, most of these variants explain only a small part of its heritability or pathogenicity. Also, CNV is the most common structural variant associated with MCPH1 (Figure 2). Often, CNVs accounted for 4.7–35% of pathogenic variants in Mendelian diseases [28].

VEP determines the effect of genetic variants (single nucleotide polymorphisms, insertions, deletions, CNVs, or structural variants) on genes, transcripts, and protein sequence, as well as regulatory regions. In addition, it integrates SIFT and PolyPhen-2 scores for changes to protein sequence. VEP has web interphase (https://www.ensembl.org/info/docs/tools/vep/index.html). There are many variants identified in the *MCPH1* gene with relatively low minor allele frequencies. The impact of low-frequency variants in this gene at the population scale is important for its pleiotropic and heterogeneity impacts. Variants present in less than 5% of individuals are described as low-frequency variants and are known to be involved in a large number of rare Mendelian disorders [29]. However, the implication of rare variants is also pervasive in common diseases and other complex traits. Indeed, mutations in MCPH1 associated with primary microcephaly autosomal recessive, and CHI autosomal recessive are rare. Interestingly, two hits associated with primary microcephaly disease were re-identified in this study but with no information about the phenotypes of the individuals. The contribution of rare and low-frequency variants to traits is largely unexplored. In humans, genetic variants are widespread but only a few of them have been associated with specific traits and diseases. Genomic databases like gnomAD present real opportunities to identify genetic variations and their distributions in various populations. However, the genome of the underrepresented group, e.g., African genome data, is underpowered to annotate genetic variants in case–control studies, a situation that is still challenging to genomic medicine advancement in Africa [30].

The high-throughput functional genomic experiments generate genome-scale datasets that require computational analyses, by using machine learning, artificial intelligence, and statistical annotation for significance scores that reflect the experimental context; many novel important findings have been achieved from the functional experiments data. For example, GO enrichment methods provide insight at the gene set level. Nonetheless, the physiological function of most genes in the human genome remains unknown. The aim of querying the GO terms and other databases to analyze the mechanistic functions of MCPH1 gene is that, perhaps, some functions might have been assigned to some genes in the GO database but not being utilized in prioritizing certain genes in genetics studies of rare diseases or unknown diagnoses. The mechanistic role of MCPH1 in DNA repair can involve a multi-enzyme and multi-pathway system to ensure the integrity of the cellular genome [31]. Harmful metabolic by-products, reactive oxygen species, environmental chemicals, and radiation largely cause DNA damage in living organisms. In this study, it is observed from the databases queried for robust information that the DNA repair machinery utilizes several different pathways to restore the genome. When the DNA cannot be repaired, the DNA repair machinery attempts to minimize the harm to ensure cell viability; however, the accumulation of DNA alterations unable to be repaired has been linked to many diseases. The seven main pathways for human DNA repair are DNA damage bypass, DNA damage reversal, base excision repair, nucleotide excision repair, mismatch repair, repair of double-strand breaks (DSBs), and repair of interstrand crosslinks (Fanconi anemia pathway). DNA repair pathways are intimately associated with other cellular processes such as DNA replication, DNA recombination, cell cycle checkpoint arrest, and apoptosis [32,33].

**Figure 5:**
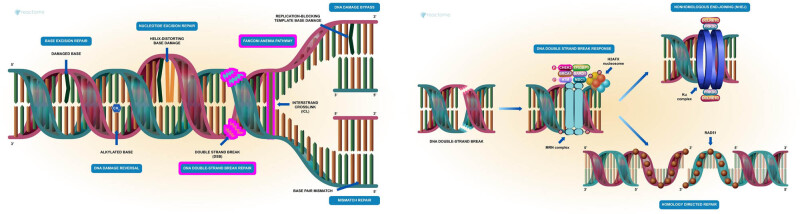
The two highlighted regions in purple show where MCPH1 functions. The MCPH1 forms parts of the MRN complex, in conjunction with SET, condensin II, SMC2, SMC4, *NCAPD3*, *NCAPH2*, and *NCAPG2*. The NHEJ pathway is initiated in response to the formation of DNA DSBs induced by DNA-damaging agents, such as ionizing radiation. The DNA DSBs are recognized by the MRN complex, leading to ATM activation and ATM-dependent recruitment of several DNA damage checkpoints and repair proteins to DNA DSB sites.

The MCPH1 protein works mostly through the repair of DSBs and the repair of interstrand crosslinks as described in Figure 5. The data retrieved from Reactome are a free, open-source, curated, and peer-reviewed pathway database. The DSBs and interstrand crosslinks are among the most deleterious types of DNA damage. DSBs can occur physiologically, especially during the processes of DNA replication and meiotic exchange [32,34]. DSBs are repaired via homology-directed repair or nonhomologous end-joining (NHEJ) [34]. The MCPH1 acts in response to DNA damage by binding condensin II complex through direct interaction with NCAPG2 and possibly NCAPD3 condensin II subunits [4,35,36]. MCPH1 binding sequesters condensin II by preventing the loading of condensin II on chromatin. Simultaneous binding of MCPH1 to the SET oncogene may contribute to condensin II sequestering [37]. Some other proteins that MCPH1 interacts with, include the SMC2, SMC4, NCAPD3, NCAPH2, and NCAPG2 [9]. It is known that these proteins are among the complex forms of proteins that unhook the DNA that have covalent bonds between two DNA strands, which disables the progression of the replication fork, so that, the replication fork bypasses the unhooked strands, and the DSB formed in the process will trigger the DSB repair mechanisms. The Reactome pathway analyses of the *MCPH1* protein suggest its involvement with ATP and other proteins. ATP depletion has been associated with histone deacetylation and hair cell death in the cochlea in HL [38]. Taken together, the biological interactions of MCPH1 in chromosome condensation and neurogenesis make its association possible in various disorders, particularly neurological and HI. HI has diverse ontologies that are associated with various phenotypes like microcephaly in a standard knowledge representation of the HI concepts or terms [39]. Indeed, a change in cell division is anticipated to decrease total neuron cell number and ultimately the cerebral cortex size. A decrease of 20% in the radial and lateral dimensions of the cerebral cortex was detected in the *Mcph1*-del brains in mice, suggesting that *Mcph1* controls the development of the cerebral cortex in mice [40,41]. An eroded cerebral cortex is linked to various neurodegenerative disorders due to neuronal cell loss [42]. Neuronal phenotypic genes that coordinate the cerebral cortex functions and sizes have been previously studied in neurodegenerative disorders like Parkinson’s disease (PD) [43].

Some very important studies on mcph1 (BRIT1) in mice earlier suggested its functional roles. For example, *BRIT1* ^−/−^ mice were studied for their essential roles in mitotic and meiotic recombination DNA repair and in maintaining genomic stability. The *BRIT1* ^−/−^ mice and mouse embryonic fibroblasts (MEFs) showed hypersensitivity to γ-irradiation. *BRIT1* ^−/−^ MEFs and T lymphocytes exhibited severe chromatid breaks, while *BRIT1* ^−/−^ mice became infertile due to gonad developmental defects observed, and meiotic homologous recombination was impaired accompanied by apoptosis and tumorigenesis. Similarly, the *BRIT1* ^−/−^ mice were growth-retarded [5,7,40,41,44].

There is an explanation for a plausible biological connection between brain disorders and HL [45]. In response to DNA damage, EYA tyrosine-protein phosphatases (EYA1, EYA2, EYA3 and, by sequence similarity, EYA4) dephosphorylate tyrosine Y142 of H2AFX and allow the progression of DNA repair [46]. MCPH1 recognizes and binds diphosphorylated H2AFX, but the exact biological role of this interaction has not been elucidated [46]. Nonetheless, the limited information about the biological roles of MCPH1 could explain why to date only a few diseases have been associated with MCPH1 despite being involved in the maintenance of genome integrity.

The latest information derived from the queried databases suggests that MCPH1 may localize to nucleoplasm; however, the function of MCPH1 localization in nucleoplasm is not known. MCPH1 is major co-localized in the centrosome and associated with mitochondria in mice and humans [6,14]. MCPH1 has been reported extensively localized in centrosomes. Microcephaly-associated proteins work with ‘satellite’ proteins that congregate near the centrosome to duplicate centrioles. The satellite proteins help to recruit four microcephaly-associated proteins to the centrosome, where they are built into a ring. The microcephaly-associated proteins congregate at the centrosome in a particular order, with each protein recruiting the next one in the sequence. Once all four are in place near the centrosome, an enzyme that helps to duplicate the centrioles joins them [47]. Three MCPH proteins, CEP152, CEP135, and STIL, interact with and promote the centrosome localization of SAS4 (also known as CPAP or CENPJ). Failure to recruit SAS4 can attenuate centriole elongation and duplication [48].

In conclusion, MCPH1 is known to contribute to maintaining cell functions, particularly by repairing damaged DNA in the cell. Identifying clinically relevant mutations or rare variants with high susceptibility to disease is highly important. As demonstrated in this study using the largest publicly available genome data, we can conclude that MCPH1 poses variants with low frequencies. The identification of variants with very low allele frequency (MAF ≤ 0.001) that are not currently associated with a phenotype suggests that the screening for these variations or closely positioned deleterious variants in MCPH1 association syndrome is reasonable. These variants are positioned in the MCPH1 most active domain and effective in consensus transcripts in the major isoform. The effect of low-frequency variants at a population scale and on a large phenotypic spectrum promotes a better understanding of genetic architecture and phenotypic variations across different populations. Nonetheless, little is still known about the MCPH1 biology. Genetic studies can unravel MCPH1 polymorphisms, heritability, and phenotypes as well as its biological connections to other related proteins.
